# The relationship of chemokine levels and the type of symptoms caused by NSAIDs or alcohol in patients with NSAIDs-exacerbated respiratory disease

**DOI:** 10.3389/fimmu.2025.1722171

**Published:** 2026-01-07

**Authors:** Karolina Frachowicz-Guerreiro, Adrian Gajewski, Maciej Chałubiński, Aleksandra Wardzyńska

**Affiliations:** Department of Immunology and Allergy, Medical University of Lodz, Lodz, Poland

**Keywords:** asthma, N-ERD, alcohol, chemokines, biomarkers

## Abstract

**Introduction:**

Nonsteroidal anti-inflammatory drug (NSAID)-exacerbated respiratory disease (N-ERD) is an asthma phenotype with a complex pathogenesis involving chemokine-mediated inflammation. This study aimed to evaluate the association between chemokine levels and symptoms triggered by NSAIDs and alcohol in patients with N-ERD.

**Methods:**

Seventy-two subjects (41 N-ERD, 20 NSAID-tolerant asthma [NTA], and 11 healthy controls) were assessed via questionnaire, lung function tests, and measurement of selected chemokines in serum, urine, and exhaled breath condensate (EBC).

**Results:**

N-ERD patients exhibited significantly higher CCL17/TARC levels in EBC compared to controls (p<0.001). Both N-ERD and NTA groups had elevated serum CCL5/RANTES levels relative to controls (p=0.002 and p=0.04, respectively). Respiratory symptoms after alcohol consumption were reported by 48% of N-ERD patients, significantly more frequent than in controls (p<0.05), with dyspnea more common in N-ERD than NTA (p=0.03). In N-ERD patients, serum CCL17/TARC levels were lower in those experiencing dyspnea after NSAIDs (p=0.03), while serum CXCL10/IP-10 levels were higher in patients with mixed (not only respiratory) symptoms (p=0.015). Alcohol symptoms in N-ERD correlated with lower urinary CCL5/RANTES levels (p=0.01). Nasal blockage after alcohol was associated with reduced CCL17/TARC in EBC (p=0.01), and dyspnea after alcohol correlated with lower serum CCL26/Eotaxin-3 (p=0.047).

**Conclusion:**

Chemokine profiles differ according to symptom type following NSAID or alcohol exposure in N-ERD patients. These findings highlight the heterogeneity of N-ERD and suggest distinct inflammatory pathways linked to clinical presentations.

## Introduction

Non-steroidal inflammatory drug (NSAID) -exacerbated respiratory disease (N-ERD) is a well-recognized asthma phenotype, traditionally described as a combination of three conditions: asthma, chronic rhinosinusitis with nasal polyps (CRSwNP) and NSAID hypersensitivity (1–3).

The pathophysiology of N-ERD involves two major mechanisms: overproduction of cysteinyl leukotrienes and increased type 2 inflammation, both significantly influenced by genetic factors and dysregulation of arachidonic acid metabolism (4). Several studies have demonstrated the essential role of chemokines in N-ERD as factors controlling the influx of innate immune cells into the airways (5–7). This superfamily of low molecular weight proteins acts as potent chemoattractants, selectively recruiting specific subsets of inflammatory cells to sites of inflammation (8). Chemokines are classified based on their structural motifs and receptor selectivity, which determine their target immune cell subsets. Eotaxins (CCL11, CCL26) primarily act via CCR3 to recruit and activate eosinophils, driving the eosinophilic inflammation characteristic of asthma and N-ERD. RANTES (CCL5), acting through CCR1, CCR3, and CCR5, exhibits broader cell tropism, attracting eosinophils, T cells, basophils, and monocytes across both acute and chronic inflammatory phases. In contrast, IP-10 (CXCL10) signals through CXCR3 to recruit activated Th1 lymphocytes, representing a distinct, non-eosinophilic inflammatory pathway (5). These chemokines thus differ principally in receptor engagement and target cell specificity, reflecting their divergent roles in N-ERD-associated immune responses.

Studies from various countries indicate a high prevalence of alcohol hypersensitivity in N-ERD patients, primarily manifesting as respiratory symptoms (9, 10). Some authors suggest that this feature may support the diagnosis of N-ERD, though the pathophysiology of this phenomenon remains unclear (1). The hypothesis posits that the intensity of inflammation in the airways, associated with the accumulation of innate cells such as eosinophils and mast cells, influences both the occurrence and severity of these reactions. On the other hand, clinical observations indicate that treatments like leukotriene modifiers, antihistamines, aspirin therapy following desensitization (ATAD), or biologics improve alcohol tolerance in individuals who previously exhibited symptoms upon exposure (10).

In this research, we evaluated specific chemokines that play a crucial role in activating inflammatory cells, which are involved in the pathophysiology of N-ERD. This pilot study focused on assessing these chemokines in biological samples obtained from individuals with N-ERD. Furthermore, we examined the correlation between the concentrations of these chemokines and the clinical features of the patients, including the types of symptoms they experience following exposure to NSAIDs or alcohol.

## Patients and study design

The study was observational in nature and all procedures were performed during a single visit The study participants were recruited between 2019 and 2022.We included 41 patients with N-ERD, 20 with asthma-tolerating NSAIDs (NSAIDs tolerant asthma, NTA), and 11 controls. Bronchial asthma was identified according to the GINA 2019 criteria3, and CRS according to the EPOS (11). N-ERD was diagnosed according to the EAACI guidelines (2) and 20 of 41 patients had diagnosis confirmed with an aspirin challenge in the past. N-ERD and NTA patients do not differ in the frequency of CRS, atopy or allergic rhinitis as well as in asthma control or severity. The control group was matched for age and sex with other groups and consisted of individuals who showed no symptoms of respiratory disease and had not been diagnosed with any chronic respiratory disorder.

Patients taking medications were asked to discontinue nasal steroids, montelukast and antihistamines seven days before the study and oral steroids 10 days before the visit. Inhaled steroids were discontinued 24 h before the visit.

Exclusion criteria included symptoms of infection or exacerbation of asthma/CRS within the past 4 weeks, malignancies, prior organ transplant, immunosuppressive therapy, biological treatment within the past 6 months, allergen immunotherapy within the past 5 years, alcohol or drug addiction, pregnancy, inability to communicate with the investigator or to understand the purpose of the study.

In all subjects from the above-mentioned group, the following tests were conducted: a questionnaire, ACT (Asthma Control Test), SNOT (Sino-Nasal Outcome Test 22), pulmonary function tests (spirometry and fractional exhaled nitric oxide, FeNO, measurement), and skin prick tests with a panel of inhalant allergens.

The questionnaire included questions regarding demographic data and the patient’s detailed medical history. Questions about past reactions to NSAIDs and alcohol covered the time of first occurrence, symptoms, the exact time from exposure to the appearance of symptoms, as well as the type of alcohol that triggered the reaction and whether, due to these reactions, the patient had limited alcohol consumption.

Venous blood samples were collected in anticoagulant tubes (Sarstedt, Germany) to determine differential blood counts and in tubes with clot activator to obtained serum (Sarstedt, Germany).

Samples intended for serum collection were first gently homogenized, set aside for 60 minutes, and centrifuged at 3,100 rpm for 15 min at 20°C. The samples were stored at -80 degrees before analysis.

Urine samples were collected from all patients. After sample delivery, the urine was centrifuged at 1,800 rpm for 10 min at 4°C. The supernatants were stored at -80 degrees before analysis.

Exhaled breath condensate (EBC) samples were collected using a TURBODECCS 09 unit (Medivac, Parma, Italy) according to the manufacturer’s instructions and ERS/ATS recommendations. Patients were asked to breathe freely for 15 minutes, until a volume of at least 1 ml was obtained. Respiratory samples were stored immediately at −80°C and kept frozen until analysis.

### Chemokines measurement

Chemokine concentrations were measured in serum using enzyme-linked immunosorbent assays (ELISA) following the manufacturer’s instructions, including CCL5/RANTES (R&D Systems, Minneapolis, MN, USA), CXCL10/IP-10 (R&D Systems), CCL17/TARC (R&D Systems), CCL22/MDC (R&D Systems); CCL11/Eotaxin-1 (R&D Systems); CCL26/Eotaxin-3 (R&D Systems). Only chemokines detected above the lower limit of detection (LLD) in at least 80% of samples (for each material) were analyzed.

### Blood eosinophilia

Cell counts and differentiation were determined using an XN-1000 hematology analyzer (Sysmex, Japan).

### Statistical analysis

Categorical variables were compared using the χ2 coefficient. Quantitative variables are presented as median and 25-75% and were compared using the Mann-Whitney U test or the Kruskal-Wallis test for multiple comparisons. Statistical analysis was performed using the Statistica software (StatSoft, Tulsa, OK, USA). Statistical significance was set at P <0.05. The correlations were calculated using Spearman’s test.

The study was approved by the local Bioethics Committee (number RNN/205/19/KE), and all study participants provided informed consent.

## Results

### Clinical characteristic of study subjects

The FEV1% predicted in the N-ERD group was similar to that in NSAID-tolerant asthma patients (**Table 1**). Similarly, there were no differences in FeNO levels or blood eosinophilia between the two groups of patients with asthma. The level of disease control assessed by the ACT or number of asthma exacerbations in the last 12 months was comparable in the N-ERD and NTA subjects. In addition, there was no significant difference in the severity of CRS symptoms measured using SNOT between the N-ERD and NTA subjects. However patients with N-ERD had a higher incidence of CRSwNP (33 (80.49%) vs 9 (45%); p=0.006) and used oCS more frequently (5 (12.20%) *vs* 0 (0%); p=0.04).

**Table 1:** Comparison of clinical and demographic characteristic of the studied groups.

Feature	N-ERD, n=41	NTA, n=20	Control, n=11	N-ERD *vs*. NTA	N-ERD *vs*. Control	NTA *vs*. Control
Age, median (25-75%)	59 (48–66)	62.5 (51.5-67.5)	58 (48–62)	ns	Ns	Ns
Female, n (%)	33 (80.49%)	12 (60%)	10 (90.91%)	ns	Ns	Ns
Asthma duration, median (25-75%)	22 (14-28.5)	15 (12-19)	NA	ns	Ns	NA
Asthma exacerbation <12 months, n (%)	28 (68.29%)	9 (45%)	0 (0%)	ns	Ns	NA
Number of exacerbations last year, median (25-75%)	1 (0-4)	0 (0-2)	NA	ns	Ns	NA
CRS, chronic sinusitis, n (%)	35 (85.37%)	15 (75%)	0 (0%)	ns	<0.001	<0.001
CRSwNP n (%)	33 (80.49%)	9 (45%)	0 (0%)	0.006	<0.001	0.002
Polypectomy, n (%)	23 (56.1%)	3 (15%)	0 (0%)	0.001	<0.001	Ns
Number of polypectomy, median (25-75%)	1 (1-3)	2 (1-3)	NA	ns	0.01	Ns
Allergic rhinitis, n (%)	25 (60.98%)	10 (50%)	1 (9.09%)	ns	0.001	0.015
Atopy, n (%)	23 (56.10%)	9 (45%)	1 (9.09%)	ns	0.005	Ns
Smoking, n (%)	3 (7.32%)	3 (15%)	3 (27.27%)	ns	Ns	Ns
Inhaled GCS, n (%)	38 (92.68%)	18 (90%)	0 (0%)	ns	<0.001	<0.001
Oral GCS, n (%)	5 (12.2%)	0 (0%)	0 (0%)	0.04	Ns	Ns
Asthma severity (mild)*, n(%)	2 (4.9%)	3 (15%)	NA	ns	NA	NA
Asthma severity (moderate)*, n(%)	4 (9.7%)	1 (5%)	NA	ns	NA	NA
Asthma severity (severe)*, n(%)	35 (85.4%)	16 (80%)	NA	ns	NA	NA
Intranasal GCS, n (%)	27 (65.85%)	11 (55%)	0 (0%)	ns	<0.001	<0.001
Blood eosinophils, median (25-75%)	0.34 (0.15-0.47)	0.23 (0.15-0.42)	0.1 (0.07-0.15)	ns	0.003	0.02
SNOT, median (25-75%)	43 (25-60)	39 (17-42)	NA	ns	NA	NA
FeNO [ppb], median (25-75%)	18 (14-38)	36.5 (24-28)	13.5 (9.5-17.5)	ns	Ns	0.02

*Asthma severity was established based on level of treatment, according to GINA (3).

N-ERD, non-steroidal anti-inflammatory drug exacerbated respiratory disease; NTA, NSAIDS-tolerant asthma; CRS, chronic sinusitis; CRSwNP, chronic sinusitis with nasal polyps; ACT, Asthma Control Test; GCZ, glucocorticoids; FEV1%pred- forced expiratory volume in 1 s percent of predicted value percent of predicted value; SNOT, sinonasal outcome test; NA, not applicable; NS, not significant; FeNO, fractional exhaled nitric Oxide.

### Chemokines levels in study group

Compared to the control group, N-ERD patients had higher levels of CCL17/TARC in EBC (1.71 pg/mL (1.55-1.84) *vs*. 1.39 (1.27-1.58); p<0.001) (**Table 2**). Both N-ERD and NTA patients had higher serum levels of CCL5/RANTES than those in the control group (respectively 534.04 (456.92-587.14) *vs*. 403.86 (396.76-455.02); p=0.002 and 471.8 (416.74-607.86) *vs*.403.86 (396.76-455.02); p=0.04). However, there were no statistically significant differences in chemokine levels between patients with N-ERD and NTA.

**Table 2:** Comparison of chemokine levels in studied groups.

Chemokine concentration [pg/mL]	N-ERD, n=41	NTA, n=20	Control, n=11	P value,	P value,	P value,
				N-ERD *vs*. NTA	N-ERD *vs*. control	NTA *vs*. control
CCL17/TARC (EBC)	1.71 (1.55-1.84)	1.58 (1.46-1.71)	1.39 (1.27-1.58)	ns	<0.001	Ns
CCL26/Eotaxin-3 (EBC)	7.74 (4.71-11.6)	9.99 (6.57-13.18)	7.74 (6.57-7.74)	ns	Ns	Ns
CCL5/RANTES (serum)	534.04 (456.92-587.14)	471.8 (416.74-607.86)	403.86 (396.76-455.02)	ns	0.002	0.04
CXCL10/IP-10 (serum)	54.17 (38.37-82.71)	57.17 (45.7-73.83)	48.14 (31.51-57.77)	ns	Ns	Ns
CCL17/TARC (serum)	79.31 (52.72-146.2)	80.49 (66.24-107.28)	63.41 (44.86-90.6)	ns	Ns	Ns
CCL22/MDC (serum)	724.96 (610.29-888.65)	721.27 (657.21-881.86)	872.15 (784.84-1188.83)	ns	Ns	Ns
CCL11/Eotaxin-1 (serum)	111.13 (86.9-129.34)	124.79 (100.39-146.78)	116.71 (78.93-146.01)	ns	Ns	Ns
CCL26/Eotaxin-3 (serum)	32.84 (29.96-43.97)	34.18 (30.19-66.78)	32.67 (26.39-40.65)	ns	Ns	Ns
CCL5/RANTES (urine)	4.98 (4.44-6.36)	5.52 (4.57-6.5)	5.52 (4.44-7.5)	ns	Ns	Ns
CCL26/Eotaxin-3 (urine)	2.43 (2.06-2.81)	2.24 (1.69-2.81)	2.43 (2.06-3.19)	ns	Ns	Ns

N-ERD, non-steroidal anti-inflammatory drug exacerbated respiratory disease; NTA, NSAIDS tolerant asthma; EBC, Exhaled breath condensate; NS, not significant.

### Associations between clinical features and chemokine levels in NTA patients

**Figure 1:**
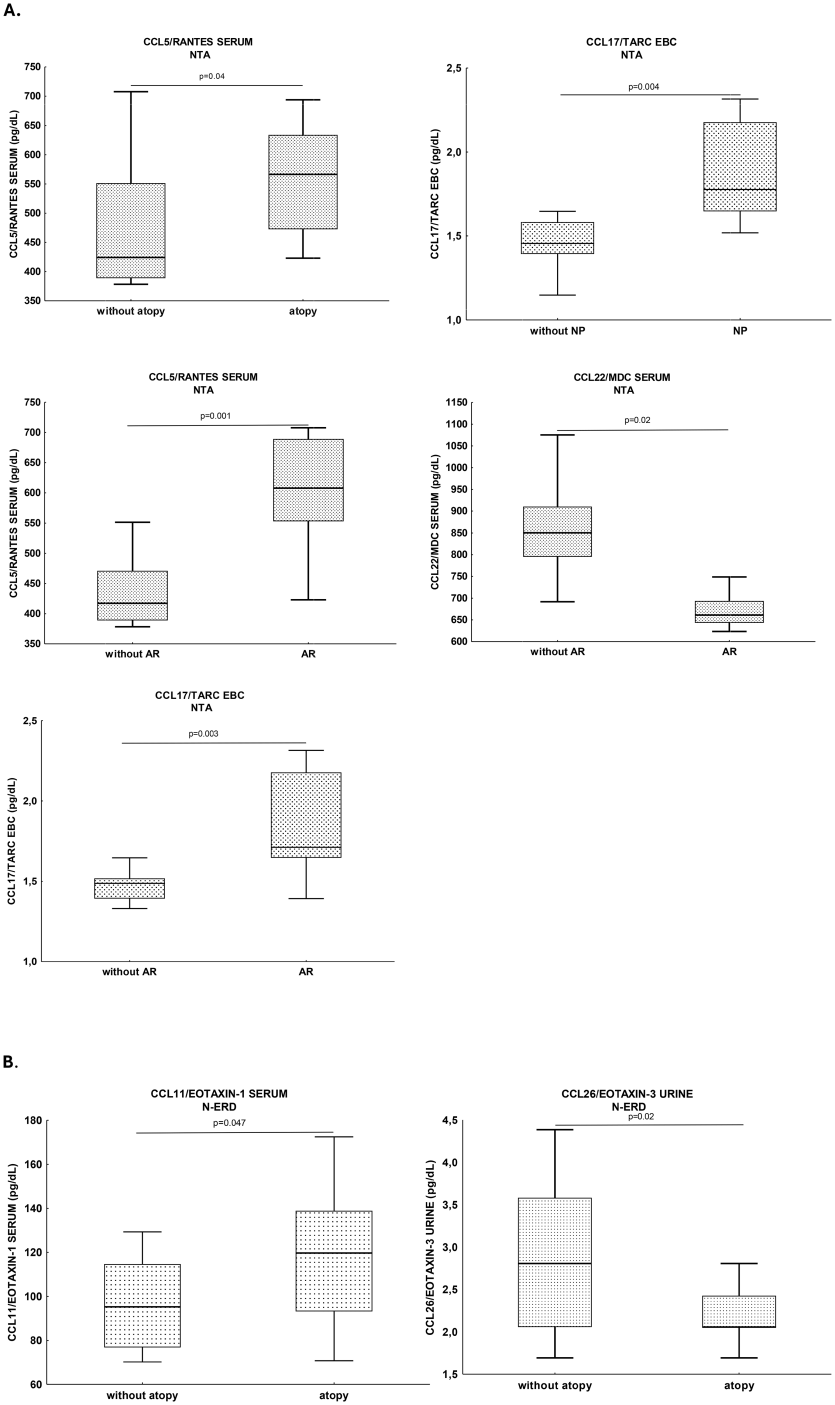
Association of chemokine levels with clinical characteristic of the subjects in **(A)** NSAIDs tolerant asthma (NTA) group **(B)** N-ERD patients. N-ERD, non-steroidal anti-inflammatory drug-exacerbated respiratory disease; NTA, non-steroidal anti-inflammatory drug-tolerant asthma; AR, allergic rhinitis; NP, nasal polyps.

In NTA patients, serum CCL11/Eotaxin-1 levels were positively correlated with age at CRS diagnosis (r=0,61, p=0.02) (**Table 3**). CCL17/TARC in the serum was negatively correlated with SNOT (r=-0,47, p=0.04). Serum CXCL10/IP-10 increased with age in the NTA group (r=0,62, p=0.004). CCL5/RANTES levels were positively correlated with SNOT (r=0,52, p=0.02) and CCL26/Eotaxin-3 levels were negatively correlated with ACT (r=-0,46, p=0.04). Patients with NTA and atopy had higher serum CCL5/RANTES levels (566.4 (472.5- 634.1) *vs*. 424.2 (388.8-551.5), p=0.04) (**Figure 1A**). Patients with CRSwNP had higher levels of CCL17/TARC in EBC (1.8 (1.6-2.2) *vs*. 1.5 (1.4-1.6), p=0.004). Patients in this group with concurrent AR had higher levels of CCL17/TARC in EBC (1,7 (1.6-2.2) *vs*. 1.5 (1.4-1.5), p=0.003) CCL5/RANTES in serum (607.1 (643.2-693.7) *vs*. 417.3 (388.8-471.1); p= 0.001) and lower levels of CCL22/MDC in serum (661.1 (643.2-693.7) *vs*. 850.5 (795.2-910.5) p=0.02).

**Table 3:** Correlations between demographic and clinical features and chemokine levels in study subjects.

Feature	Serum				Urine	
	CCL11/Eotaxin-1	CCL17/TARC	CCL26/Eotaxin-3	CXCL10/IP-10	CCL5/RANTES	CCL26/Eotaxin-3
N-ERD						
Age of diagnosis of polyps	ns	ns	-0.38, p=0.03	Ns	ns	ns
Number of episodes after NSAIDs	ns	ns	0.33, p=0.03	Ns	ns	ns
Blood eosinophils	ns	0.32, p=0.04	Ns	Ns	ns	ns
FEV1%pred	ns	-0.37, p=0.02	Ns	Ns	ns	ns
NTA						
Age	ns	ns	Ns	0.62, p=0.004	ns	ns
Age of diagnosis of CRS	0.61, p=0.02	ns	Ns	Ns	ns	ns
ACT	ns	ns	Ns	Ns	ns	-0.46, p=0.04
SNOT	ns	-0.47, p=0.04	Ns	Ns	0.52, p=0.02	ns

N-ERD, non-steroidal anti-inflammatory drug exacerbated respiratory disease; NTA, non-steroidal anti-inflammatory drug tolerant asthma; ACT, Asthma Control Test; FEV1%pred, forced expiratory volume in 1 s percent of predicted value percent predicted; CRS, chronic rhinosinusitis; SNOT, SNOT-sinonasal outcome test 22; NS, not significant.

### Associations between clinical features and chemokine levels in N-ERD patients

In patients with N-ERD, serum CCL17/TARC levels were positively correlated with the number of EOS (r= 0.32, p=0.04) and negatively correlated with FEV1 (r-0.37, p-0.02) (**Table 3**). Serum CCL26/Eotaxin-3 levels was positively correlated with the number of reactions to NSAIDs (r=0.33, p=0.03) and negatively with the age of diagnosis of nasal polyps (r=-0.38, p=0.03). Patients with N-ERD who had atopy had higher levels of CCL11/Eotaxin-1 in the serum (119.7 (93.2-138.9) *vs* 95.2 (76.8-114.7), p=0.047) and lower CCL26/Eotaxin-3 in the urine (2.1 (2.1-2.4) *vs*. 2.8 (2.1-3.6), p=0.02) (**Figure 1B**).

### Characteristic of NSAIDs induced symptoms in N-ERD patients

The most common type of reaction to NSAIDs was bronchial symptoms, which were reported by 37 (90.24%) patients with N-ERD (**Table 4**). Of the non-respiratory reactions, the most common was a fainting/blood pressure (BP) drop in 18 (43.9%) subjects, followed by skin symptoms in 17 (41.46%). Mixed symptoms after NSAIDs administration (from the respiratory and cardiovascular and/or gastrointestinal system) were present in 28 (68.29%) patients with N-ERD.

**Table 4:** Characteristic of NSAIDs related symptoms in N-ERD patients.

Feature	n=41
Bronchial symptoms	37 (90.24%)
Nasal symptoms	18 (43.9%)
Bronchial or nasal symptoms	41 (100%)
Decrease in BP	18 (43.9%)
GI symptoms	8 (19.51%)
Skin symptoms	17 (41.46%)
Mixed symptoms	28 (68.29%)

N-ERD, non-steroidal anti-inflammatory drug exacerbated respiratory disease; BP,- blood pressure; GI, gastrointestinal.

**Figure 2:**
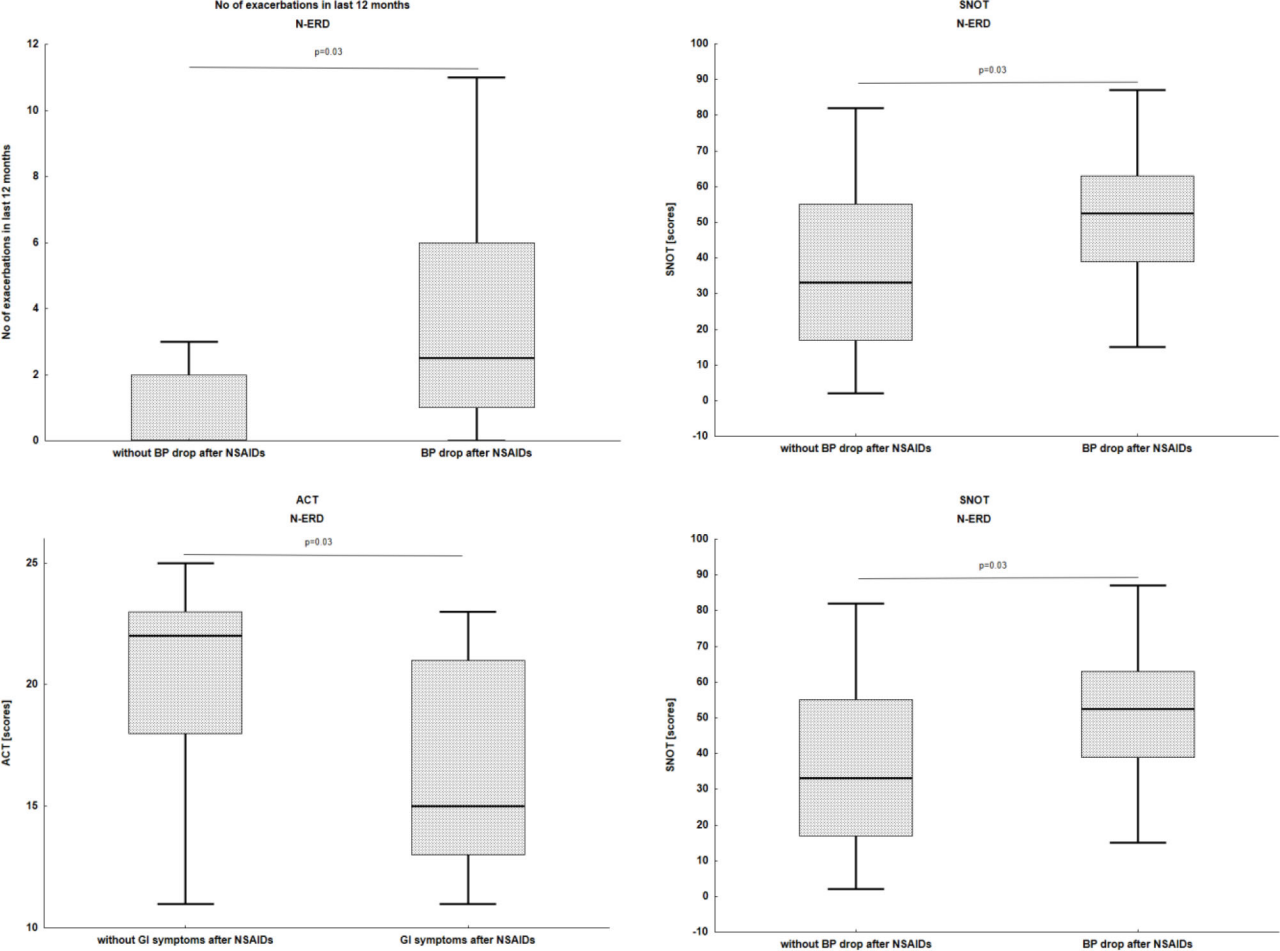
Association of clinical characteristic with types of reactions after NSAIDs exposure in N-ERD patients. N-ERD, non-steroidal anti-inflammatory drug exacerbated respiratory disease; NSAIDs, non-steroidal anti-inflammatory drugs; BP, blood pressure; GI, gastrointestinal; SNOT, sinonasal outcome test 22. The box represents the 25th–75th percentile (interquartile range), the horizontal line indicates the median, and the whiskers show the range of non-outlier values.

Patients who reported a fainting/BP drop after NSAIDs had a higher number of asthma exacerbations in the past 12 months (3 (1–6) *vs*. 1 (0–2), p= 0.03) and worse control of upper airway symptoms measured as higher SNOT score (53 (43–63) *vs*. 37 (19–55), p=0.03) (**Figure 2**). Patients with N-ERD who had GI symptoms after NSAIDs administration had lower ACT score (15 (14–22) *vs* 21 (18–23), p=0.03) and higher SNOT score (62 (53–73) *vs*. 38 (19–59), p=0.002).

### Characteristic of alcohol induced symptoms in N-ERD patients

Symptoms after alcohol consumption were present in 20 (48%) patients with N-ERD, 7 (35%) patients with NTA, and only in 1 (9%) subject from control group (**Table 5**). Dyspnea was more frequent in patients with N-ERD than in those with NTA (p=0,03) (**Table 5**).

**Table 5:** Characteristic of alcohol related symptoms in N-ERD and NTA patients.

Feature	N-ERD. n=41	NTA. n=20	N-ERD *vs*. NTA
Any symptoms	20 (48.78%)	7 (35%)	Ns
Nasal blockage	10 (24.39%)	3 (15%)	Ns
Watery discharge	12 (29.27%)	5 (25%)	Ns
Any nasal symptoms	15 (36.59%)	5 (25%)	Ns
Shortness of breath	11 (26.83%)	1 (5%)	0.03
Wheezing	6 (14.63%)	1 (5%)	Ns
Any bronchial symptoms	12 (29.27%)	2 (10%)	Ns
Any nasal or bronchial symptoms	19 (46.34%)	5 (25%)	Ns
Skin symptoms	5 (12.2%)	2 (10%)	Ns

N-ERD, non-steroidal anti-inflammatory drug exacerbated respiratory disease; NTA, non-steroidal anti-inflammatory drug tolerant asthma; NS, not significant.

Interestingly, of the 17 N-ERD patients who reported not only but also skin symptoms after NSAIDs administration, only three had nasal symptoms after alcohol consumption (p=0.03).

In addition, among the eight patients with N-ERD who reported additional abdominal symptoms after taking NSAIDs, none reported nasal symptoms after alcohol use (p=0.01).

### Associations between NSAIDs or alcohol induced symptoms and chemokine levels in N-ERD patients

**Figure 3:**
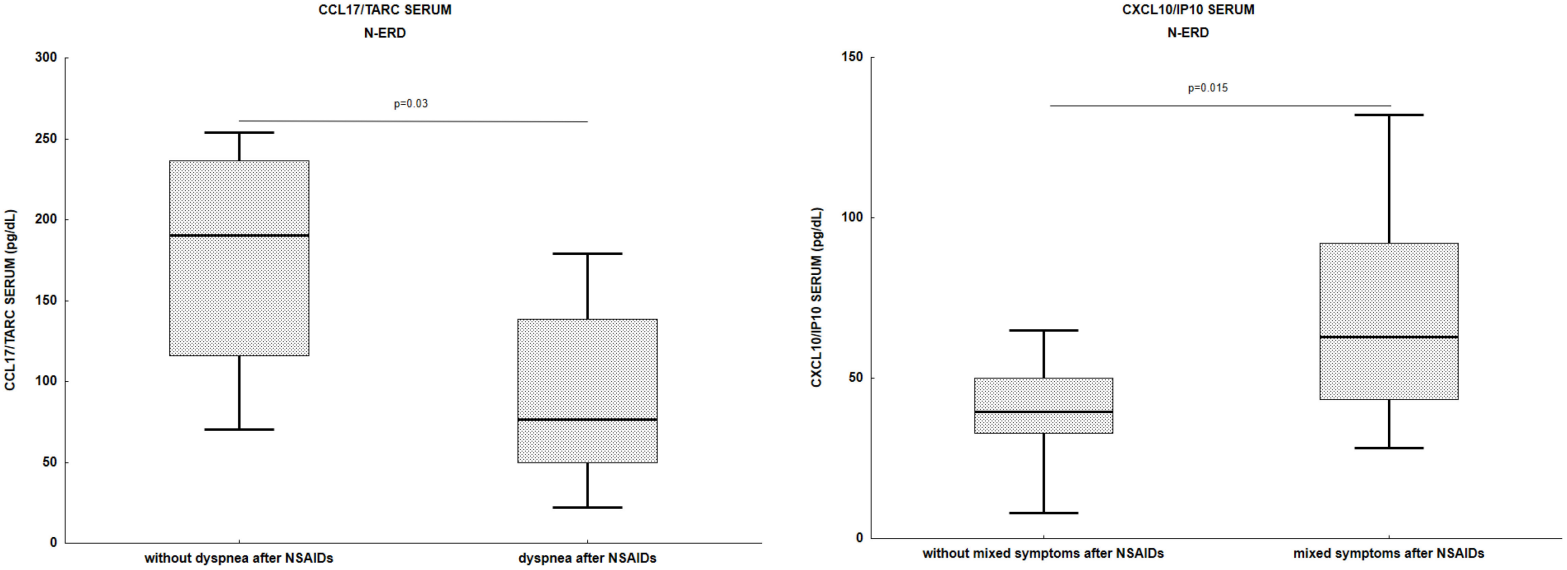
Association of chemokines level with types of reactions after NSAIDs exposure in N-ERD patients. N-ERD, non-steroidal anti-inflammatory drug exacerbated respiratory disease; NSAIDs, non-steroidal anti-inflammatory drugs.

Serum CCL17/TARC levels were lower in patients with dyspnea after NSAIDs administration (76.5 (49.9-139.7) *vs*. 150.1 (70.2-218.6) p=0.03) (**Figure 3**). In contrast, serum CXCL10/IP10 levels were higher in patients who had mixed (not only respiratory) symptoms after NSAIDs administration (57.7 (38.4-82.1) *vs*. 49 (43-77.7), p=0.015).

**Figure 4:**
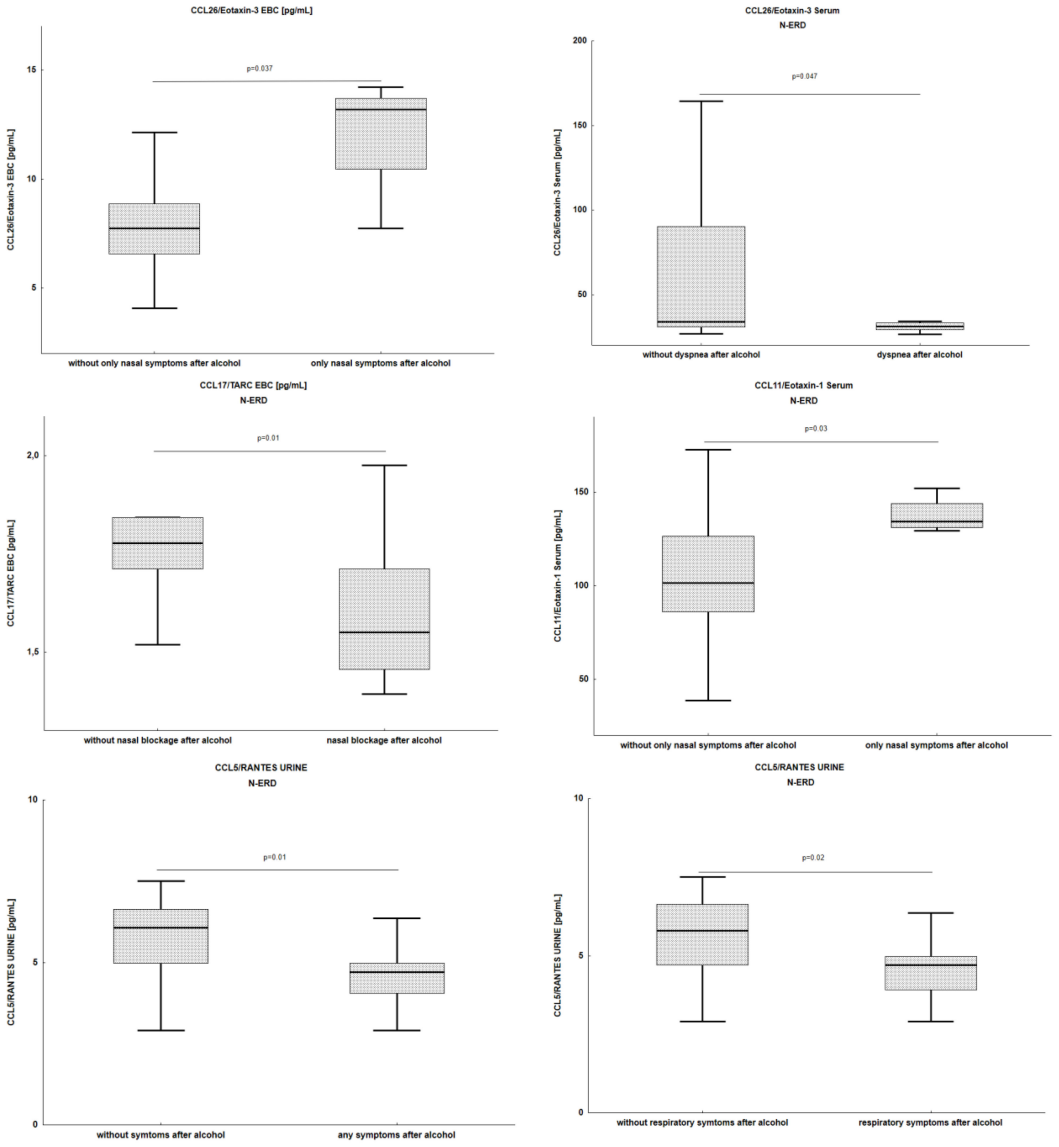
Association of chemokines level with types of reactions after alcohol consumption in N-ERD patients. N-ERD, non-steroidal anti-inflammatory drug exacerbated respiratory disease.

Occurrence of any post-alcohol symptoms in patients with N-ERD were associated with lower CCL5/RANTES levels in urine (4.7 (4–5) *vs*. 6.1 (5-6.3), p=0.01) (**Figure 4**). Similarly, CCL5/RANTES levels were also lower in the urine of patients who reported respiratory symptoms after alcohol consumption (4.7 (3.9-5) *vs*. 6 (4.7-6.6), p=0.02). Nasal blockage after alcohol consumption was associated with lower levels of CCL17/TARC in EBC (1.6 (1.5-1.7) *vs*. 1.8 (1.7-1.8), p=0.01), and dyspnea after alcohol consumption was associated with lower levels of CCL26/Eotaxin-3 in the serum (31.1 (29.2-33.2) *vs*. 33.9 (30.9-90.1), p=0.047). Patients who reported only nasal symptoms after alcohol consumption had higher levels of CCL26/Eotaxin-3 in EBC (13.2 (10.5-13.7) *vs*. 7.7 (6.6-8.8), p=0.037) and CCL11/Eotaxin-1 in serum (134.1 (131.-143.7) *vs*. 101.4 (85.8-126.3), p=0.03).

## Discussion

In this pilot study involving patients with N−ERD, NSAID−tolerant asthma (NTA), and healthy controls, systemic chemokine profiles did not differentiate N−ERD from NTA. However, these levels varied only when compared to healthy individuals. Some authors have shown a different expression of chemokines in patients with N-ERD compared to that in asthmatics who tolerate NSAIDs (12). A potential mechanism of action of chemokines involves the engagement of these mediators in chronic inflammation rather than during the initiation of acute symptoms caused by NSAIDs. A study by Kupczyk et al. (13) showed that CCL5/RANTES and MCP-3 were responsible for the influx of eosinophils in the chronic phase of inflammation, but not after ASA administration. Interestingly, in N-ERD patients treated with dupilumab, an antibody against IL-4/13, a decrease in CCL11/Eotaxin-1, CCL26/Eotaxin-3 and CCL5/RANTES was observed in nasal secretions, and the levels of CCL11/Eotaxin-1 and CCL17/TARC were found to be predictors of ASA tolerance (14). The lack of differences observed in our study may be related to the fact that we selected patients from both groups according to the presence of atopy and CRS and did not assess these mediators in material from the upper respiratory tract. Although these chemokines have been shown to be associated with N-ERD, it appears that this mechanism is not specific to this disease entity (15). Interestingly, in our study, CCL17/TARC was associated with blood eosinophilia only in patients with N-ERD. CCL17/TARC is a chemokine produced by, among others, monocytes/macrophages as well as epithelial cells, which specifically regulates the recruitment of T lymphocytes. The synthesis of CCL17/TARC, depending on the site of production and can be regulated by various factors, including IL4/IL13, TNF-α and IFN-γ (16), cytokines previously indicated as being associated with the N-ERD pathogenesis (17). In contrast, we observed associations between chemokine levels and parameters that may indicate greater asthma or CRS severity in both groups of patients with asthma.

Research has shown that alcohol causes respiratory reactions in a substantial proportion of patients with N-ERDs (9). We noted that symptoms after alcohol consumption were present in 48% of patients with N-ERD, 35% with NTA, and 9% of controls. As in other studies, respiratory symptoms prevailed, with dyspnea being the most common respiratory symptom. The similar incidence of post-alcohol symptoms in the N-ERD and ATA groups may have been related to the selection in terms of severity and control of the disease, and the fact that most patients with asthma without hypersensitivity to NSAIDs had concomitant CRS. These findings are particularly noteworthy when compared with those of the general population. In a cross-sectional study from 2008 involving 4,066 participants, the rates of alcohol-induced upper and lower respiratory reactions were 7.6% and 3.2%, respectively. Even among individuals with asthma, the prevalence was lower than that among patients with N-ERD. A systematic review of the literature further substantiated the correlation between alcohol sensitivity and N-ERD. Of the 522 patients with N-ERD, 52.8% reported at least one sinopulmonary exacerbation after alcohol intake (18). Few studies have investigated the mechanism of post-alcohol symptoms in patients with N-ERD. Both mediators, mainly leukotrienes, and innate cells, mostly mast cells and eosinophils, have been suggested to be involved. In one of the few original *in vitro* studies on this topic, involving not only N-ERD but also NSAID-tolerant patients with CRS, it was shown that alcohol can cause aggregation of platelet and granulocyte complexes, which are responsible for the release of lipid mediators. Interestingly, one study has indicated that CCL22/MDC and CCL17/TARC can cause platelet aggregation and calcium efflux (19). In our group, we noticed a relationship between chemokine levels and reactions reported after alcohol consumption. Furthermore, we also observed variations according to the type of alcohol-related symptoms reported and chemokine concentrations. Recently, a new hypothesis has emerged to explain the alcohol-induced symptoms in patients with N-ERD, linking it to locally impaired alcohol metabolism within the respiratory tract. This study specifically revealed that in N-ERD patients, reduced expression of aldehyde dehydrogenase 2 (ALDH2) in the nasal epithelium, likely driven by elevated IL-4 and IL-13, leads to the accumulation toxic metabolite of ethanol, acetaldehyde. This accumulation seems to trigger mast cell activation, resulting in respiratory symptoms following alcohol consumption. Furthermore, treatment with dupilumab was shown to restore ALDH2 expression and improve alcohol tolerance in these patients. These findings provide novel mechanistic insights into the underpinnings of alcohol-related respiratory symptoms in N-ERD and suggest that targeting cytokine pathways may benefit not only chronic inflammation but also specific triggers that affect patient quality of life (10).

Recently, attention has been paid to the diversity of patients with N-ERDs. Studies using factor analysis or data mining methods that consider the clinical characteristics of patients show sub-phenotypes within the group (20). Additional data on the heterogeneity of the N-ERD patient group were provided by studies on lower airway inflammation (21, 22). In our group, we also observed variations, including hypersensitivity reactions to NSAIDs and alcohol. The above-mentioned studies showed that alcohol-induced symptoms in patients with N-ERD somehow mimic the type of hypersensitivity reaction after NSAIDs. However, the fact that these symptoms did not occur in all patients seems to be a sign of the heterogeneity of this group. Some authors also emphasize that alcohol-related symptoms do not improve in all patients with N-ERD undergoing aspirin (9) is also evidence of phenotypic variation. In this light, our observations seem interesting because we noticed that patients with mixed reactions after NSAIDs had significantly fewer upper respiratory symptoms after alcohol consumption.

Our study had several limitations. First, the study group was small, and the selection of patients was not random, but purposive. Moreover, chemokine levels were tested during the stable period of the disease, not during aspirin provocation, which could have contributed to more information. We also did not explain the mechanism of action of chemokines in the development of symptoms in the patients with N-ERD.

In summary, the dysregulation of chemokines plays a role in chronic airway inflammation in asthma with CRS, which limits their effectiveness as sole biomarkers for distinguishing N-ERD from NTA. Nonetheless, we identified variations in the frequency and nature of reactions to NSAIDs and alcohol among N-ERD patients, indicating their phenotypic diversity. Furthermore, we demonstrated that these reactions might be linked to chemokine levels in different biological samples. Future research should focus on sampling from the upper airway compartment and conducting longitudinal or provocation studies to assess the significance of chemokine profiles in the pathogenesis of N-ERD.

## Data Availability

The raw data supporting the conclusions of this article will be made available by the authors, without undue reservation.

## References

[1] Szczeklik A Nizankowska E Duplaga M . Natural history of aspirin-induced asthma. AIANE Investigators. European Network on Aspirin-Induced Asthma. Eur Respir J. (2000) 16:432–6. doi: 10.1034/j.1399-3003.2000.016003432.x 11028656

[2] Kowalski ML Agache I Bavbek S Bakirtas A Blanca M Bochenek G . Diagnosis and management of NSAID-Exacerbated Respiratory Disease (N-ERD)-a EAACI position paper. Allergy. (2019) 74:28–39. doi: 10.1111/all.13599 30216468

[3] Global Initiative for Asthma . Global Strategy for Asthma Management and Prevention (2019). Available online at: www.ginasthma.org (Accessed November 11, 2025).

[4] Lyly A Laidlaw TM Lundberg M . Pathomechanisms of AERD-recent advances. Front Allergy. (2021) :734733. doi: 10.3389/falgy.2021.734733 35387030 PMC8974777

[5] Liu C Zhang X Xiang Y Qu X Liu H Liu C . Role of epithelial chemokines in the pathogenesis of airway inflammation in asthma (Review). Mol Med Rep. (2018) 17:6935–41. doi: 10.3892/mmr.2018.8739 29568899

[6] Kalinauskaite-Zukauske V Januskevicius A Janulaityte I Miliauskas S Malakauskas K . Expression of eosinophil β chain-signaling cytokines receptors, outer-membrane integrins, and type 2 inflammation biomarkers in severe non-allergic eosinophilic asthma. BMC Pulm Med. (2019) 19:158. doi: 10.1186/s12890-019-0904-9 31438916 PMC6706886

[7] Stevens WW Ocampo CJ Berdnikovs S Sakashita M Mahdavinia M Suh L . Cytokines in chronic rhinosinusitis. Role in eosinophilia and aspirin-exacerbated respiratory disease. Am J Respir Crit Care Med. (2015) 192:682–94. doi: 10.1164/rccm.201412-2278OC 26067893 PMC4595675

[8] Baggiolini M . Chemokines in pathology and medicine. J Intern Med. (2001) 250:91–104. doi: 10.1046/j.1365-2796.2001.00867.x 11489059

[9] Cardet JC White AA Barrett NA Feldweg AM Wickner PG Savage J . Alcohol-induced respiratory symptoms are common in patients with aspirin exacerbated respiratory disease. J Allergy Clin Immunol Pract. (2014) 2:208–13. doi: 10.1016/j.jaip.2013.12.003 24607050 PMC4018190

[10] Zawacki M Huang GX Cho L Omilabu V Bensko JC Roditi RE . Reduced aldehyde dehydrogenase 2 in respiratory tract associates with dysregulated alcohol metabolism and respiratory reactions in aspirin-exacerbated respiratory disease. J Allergy Clin Immunol. (2025) 156(6):1706–14.e1. doi: 10.1016/j.jaci.2025.08.011. 40885289 PMC12442335

[11] Fokkens WJ Lund VJ Hopkins C Hellings PW Kern R Reitsma S . European position paper on rhinosinusitis and nasal polyps 2020. Rhinology. (2020) 58:1–464. doi: 10.4193/Rhin20.401 32077450

[12] Pods R Ross D van Hülst S Rudack C Maune S . RANTES, eotaxin and eotaxin-2 expression and production in patients with aspirin triad. Allergy. (2003) 58:1165–70. doi: 10.1034/j.1398-9995.2003.00276.x 14616128

[13] Kupczyk M Kurmanowska Z Kupryś-Lipińska I Bocheńska-Marciniak M Kuna P . Mediators of inflammation in nasal lavage from aspirin intolerant patients after aspirin challenge. Respir Med. (2010) 104:1404–9. doi: 10.1016/j.rmed.2010.04.017 20452758

[14] Schneider S Poglitsch K Morgenstern C Quint T Gangl K Sinz C . Dupilumab increases aspirin tolerance in NSAID-exacerbated respiratory disease. Eur Respir J. (2023) 61:2201335. doi: 10.1183/13993003.01335-2022 36549708 PMC10017890

[15] Yamada A Kiryu K Takashino S Yoshida M Takeichi T Kitamura O . Diagnostic value of serum thymus and activation-regulated chemokine (TARC) in fatal asthma. Forensic Sci Int. (2024) 365:112276. doi: 10.1016/j.forsciint.2024.112276 39486256

[16] Catherine J Roufosse F . What does elevated TARC/CCL17 expression tell us about eosinophilic disorders? Semin Immunopathol. (2021) 43:439–58. doi: 10.1007/s00281-021-00857-w 34009399 PMC8132044

[17] Frachowicz-Guerreiro K Gajewski A Ćwikliński R Kurowski M Chałubiński M Wardzyńska A . Cytokine profile in the upper airways of patients with N-ERD obtained via a minimally invasive method. J Immunol Res. (2025) 2025:2768458. doi: 10.1155/jimr/2768458 41179970 PMC12572634

[18] Candelo E McCalla M Valderrama OA Avila-Castano K Chelf C Olomu O . Relationship between alcohol intolerance and aspirin-exacerbated respiratory disease (AERD): systematic review. Otolaryngol Head Neck Surg. (2023) 169:12–20. doi: 10.1002/ohn.248 36939486

[19] Abi-Younes S Si-Tahar M Luster AD . The CC chemokines MDC and TARC induce platelet activation via CCR4. Thromb Res. (2001) 101:279–89. doi: 10.1016/s0049-3848(00)00402-3 11248289

[20] Bochenek G Kuschill-Dziurda J Szafraniec K Plutecka H Szczeklik A Nizankowska-Mogilnicka E . Certain subphenotypes of aspirin-exacerbated respiratory disease distinguished by latent class analysis. J Allergy Clin Immunol. (2014) 133:98–103.e1-6. doi: 10.1016/j.jaci.2013.07.004 23993879

[21] Celejewska-Wójcik N Wójcik K Ignacak-Popiel M Ćmiel A Tyrak K Gielicz A . Subphenotypes of nonsteroidal antiinflammatory disease-exacerbated respiratory disease identified by latent class analysis. Allergy. (2020) 75:831–40. doi: 10.1111/all.14141 31803947 PMC7216982

[22] Jakiela B Soja J Sladek K Przybyszowski M Plutecka H Gielicz A . Heterogeneity of lower airway inflammation in patients with NSAID-exacerbated respiratory disease. J Allergy Clin Immunol. (2021) 147:1269–80. doi: 10.1016/j.jaci.2020.08.007 32810516

