# Correction: The relationship of chemokine levels and the type of symptoms caused by NSAIDs or alcohol in patients with NSAIDs-exacerbated respiratory disease

**DOI:** 10.3389/fimmu.2026.1811971

**Published:** 2026-06-17

**Authors:** Karolina Frachowicz-Guerreiro, Adrian Gajewski, Maciej Chałubiński, Aleksandra Wardzyńska

**Affiliations:** Department of Immunology and Allergy, Medical University of Lodz, Lodz, Poland

**Keywords:** asthma, N-ERD, alcohol, chemokines, biomarkers

There was a mistake in **Figure 1** as published. During the production process, an incorrect version of **Figure 1** was used in the published article. The corrected **Figure 1** appears below. This correction does not affect the scientific conclusions of the original article.

**Figure 1:**
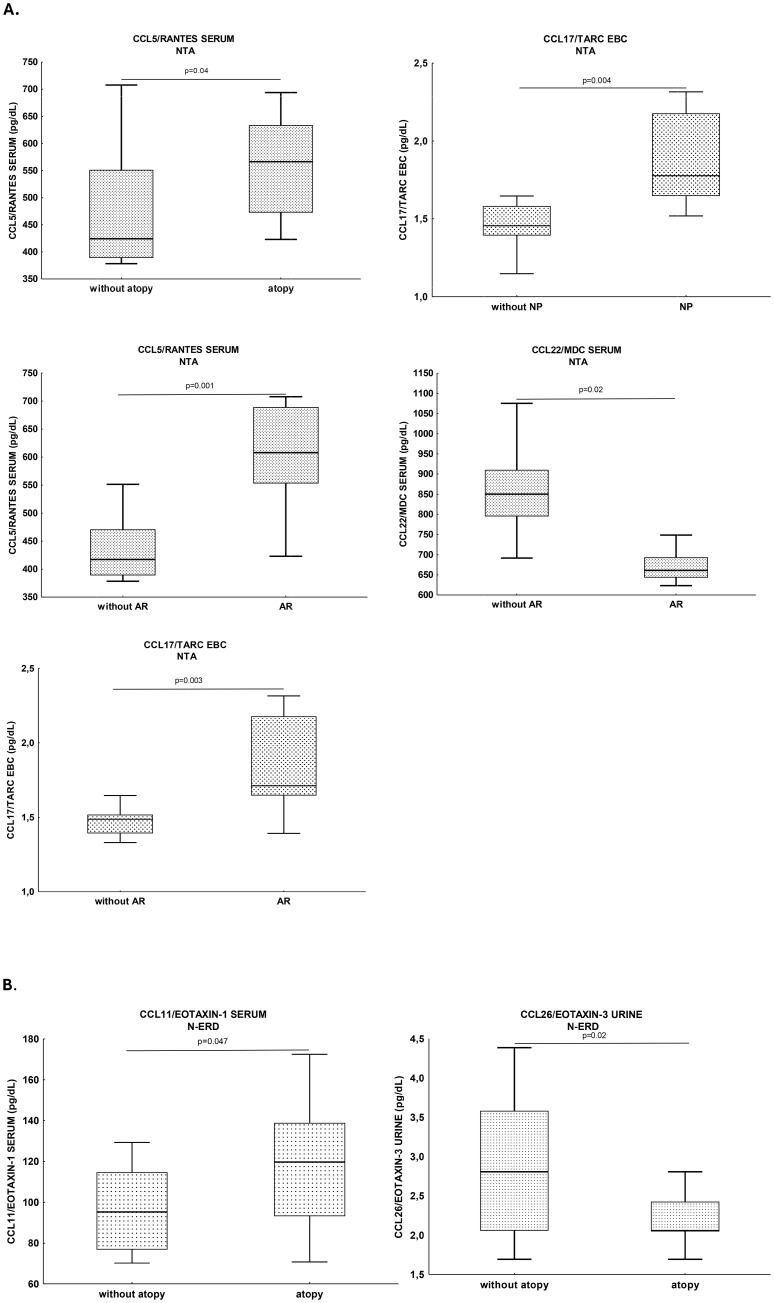
Association of chemokine levels with clinical characteristics of the subjects in **(A)** NSAIDs tolerant asthma (NTA) group **(B)** N-ERD patients. N-ERD, non-steroidal anti-inflammatory drug-exacerbated respiratory disease; NTA, non-steroidal anti-inflammatory drug-tolerant asthma; AR, allergic rhinitis; NP, nasal polyps.

The box represents the 25th–75th percentile (interquartile range), the horizontal line indicates the median, and the whiskers show the range of non-outlier values.

The original version of this article has been updated.

